# Identifying radiation responsive exon-regions of genes often used for biodosimetry and acute radiation syndrome prediction

**DOI:** 10.1038/s41598-022-13577-4

**Published:** 2022-06-09

**Authors:** Simone Schüle, Patrick Ostheim, Matthias Port, Michael Abend

**Affiliations:** Bundeswehr Institute of Radiobiology Affiliated to the University Ulm, Neuherbergstr. 11, 80937 Munich, Germany

**Keywords:** Diagnostic markers, Prognostic markers, Reverse transcription polymerase chain reaction

## Abstract

Gene expression (GE) analysis of *FDXR, DDB2, WNT3* and *POU2AF1* is a promising approach for identification of clinically relevant groups (unexposed, low- and high exposed) after radiological/nuclear events. However, results from international biodosimetry exercises have shown differences in dose estimates based on radiation-induced GE of the four genes. Also, differences in GE using next-generation-sequening (NGS) and validation with quantitative real-time polymerase chain reaction (qRT-PCR) was reported. These discrepancies could be caused by radiation-responsive differences among exons of the same gene. We performed GE analysis with qRT-PCR using TaqMan-assays covering all exon-regions of *FDXR, DDB2, WNT3* and *POU2AF1*. Peripheral whole blood from three healthy donors was X-irradiated with 0, 0.5 and 4 Gy. After 24 and 48 h a dose-dependent up-regulation across almost all exon-regions for *FDXR* and *DDB2* (4–42-fold) was found. A down-regulation for *POU2AF1* (two- to threefold) and *WNT3* (< sevenfold) at the 3’-end was found at 4 Gy irradiation only. Hence, this confirms our hypothesis for radiation-responsive exon-regions for *WNT3* and *POU2AF1*, but not for *FDXR* and *DDB2*. Finally, we identified the most promising TaqMan-assays for *FDXR* (e.g. AR7DTG3, Hs00244586_m1), *DDB2* (AR47X6H, Hs03044951_m1), *WNT3* (Hs00902258_m1, Hs00902257_m1) and *POU2AF1* (Hs01573370_g1, Hs01573371_m1) for biodosimetry purposes and acute radiation syndrome prediction, considering several criteria (detection limit, dose dependency, time persistency, inter-individual variability).

## Introduction

In case of nuclear or radiologic emergency events (e.g. dirty bomb, reactor catastrophe) with a large number of potential victims, rapidly administered and high-throughput diagnostic tests are needed to quickly and reliably differentiate unexposed (the worried well) from actual low and high exposed radiation accident victims^1^. To create and maintain radiation emergency preparedness, dose assessment exercises^2–4^ are performed regularly by European and international organizations, such as RENEB (Running the European Network of Biological and retrospective Physical dosimetry) or NATO^5–7^.

The analysis of gene expression changes (GE) shows promise for rapid administration with high-throughput diagnostic testing^8^. Specific radiation-sensitive genes (such as *FDXR, DDB2, WNT3, POU2AF1*) have become well established for biodosimetry purposes and acute radiation sickness (ARS)-prediction^9–12^. *FDXR* and *DDB2* are commonly used biomarkers for retrospective dosimetry within 72 h after radiation exposure^13–15^, especially in combination with *WNT3* and *POU2AF1* it has become possible not only to provide dose estimation, but to distinguish three groups of clinical significance (see above). Up-regulation of *FDXR* and *DDB2* GE indicates risk of mild acute radiation sickness, whereas an up-regulation of *FDXR* and *DDB2* combined with a down-regulation of *WNT3* and *POU2AF1* predicts severe ARS^10,11^.

In comparing qRT-PCR results of RENEB 2019 exercise participants, differences in *FDXR* and *DDB2* upregulation were found^16^. To explain fold-differences in up- or down-regulated genes reported by various teams we hypothesized that while TaqMan assays target the same genes, they assess different exon-pairs within that gene. These exon-pairs might be more or less responsive to radiation exposure.

Additionally, in our previous studies we sometimes experienced difficulties in validating next generation sequencing (NGS) GE changes with quantitative real-time polymerase chain reaction (qRT-PCR)^17^. This might be explained considering the conventional NGS method sums the reads across all exons of a gene and are attributed to that gene. In contrast, qRT-PCR TaqMan assays selectively recognize one specific exon-pair of one gene. The different findings with respect to GE changes might be explained depending on whether the whole gene (NGS) or only an exon-region of the gene is interrogated (qRT-PCR).

The “best coverage” TaqMan assay is recommended by the manufacturer, because it ascertains the maximum number of transcripts (increased detection limits) of the examined gene and it best meets the following criteria compared to other assays: it (1) does not identify targets with (almost) similar sequences (homologs), (2) has a short amplicon length for efficient PCR and (3) spans introns^18^. We questioned whether the "best coverage" TaqMan assay was the most suitable for investigating radiation-induced gene expression changes.

In the present study, we examined radiation-induced gene expression changes of *FDXR, DDB2, WNT3,* and *POU2AF1* at the exon level using qRT-PCR. We evaluated all exon-pairs of these genes for the most radiation-responsive region. We also considered detection limits (sufficient baseline gene copy numbers), dose dependencies, persisting gene expression over 48 h, and inter-individual variability.

## Materials and methods

### Sample collection, irradiation and cell culture

**Figure 1:**
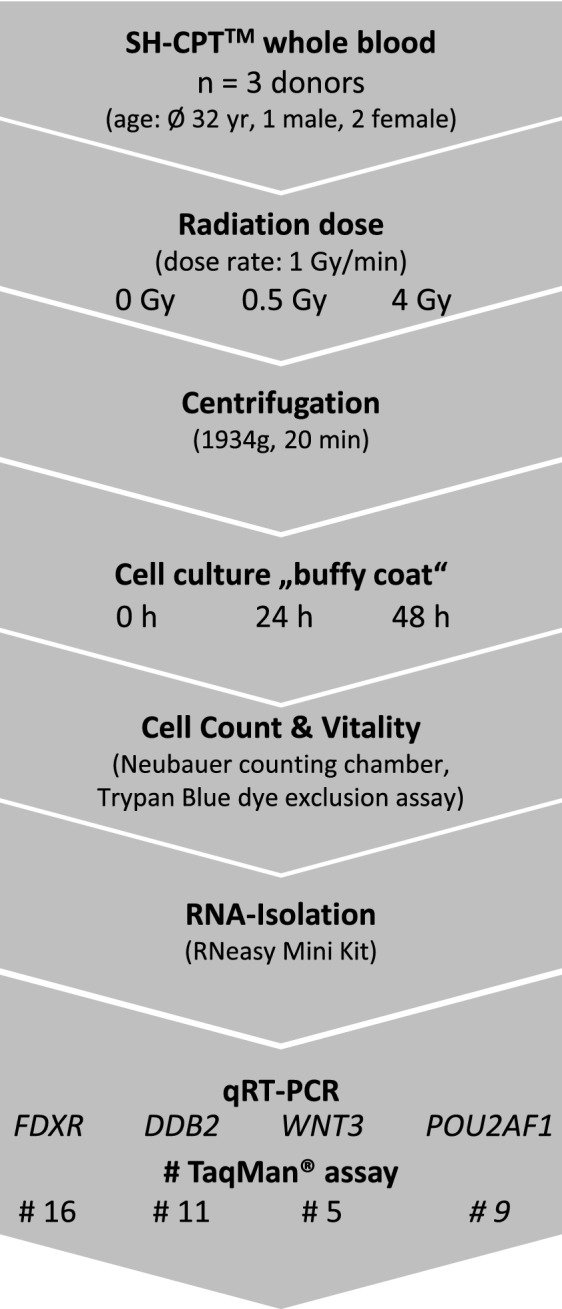
Study overview. Study design overview including X-irradiation of SH-CPT blood, centrifugation to isolate PBMCs, in vitro culture, cell count and viability measurements, RNA isolation and gene expression measurements with TaqMan assays covering all exon-regions of *FDXR, DDB2, WNT3* and *POU2AF1*. SH-CPT = Cell Preparation Tube with Sodium Heparin. PBMC = peripheral blood mononuclear cells. Ø = average age of donors. # = number of TaqMan assays.

Peripheral whole blood from three healthy donors (one male and two female volunteers, aged 23–39) was collected into Becton Dickinson (BD) Vacutainer Sodium Heparin Cell Preparation Tubes (SH-CPT) (BD, Heidelberg, Germany). The blood tubes were either sham irradiated or exposed to 0.5 or 4 Gy (Fig. 1). In vitro irradiation was performed at 37 °C using single doses of X-rays filtered with 3 mm beryllium and 3 mm aluminum to give a mean photon energy of 100 keV (Maxishot SPE cabin, Yxlon, Hamburg, Germany). The absorbed doses were measured using a UNIDOS webline 10021 dosimeter (PTW, Freiburg, Germany). The dose-rate was approximately 1.0 Gy/min at 13 mA and accelerating potential 240 kV (maximum photon energy of 240 keV). After irradiation, mononuclear cells and autologous plasma were separated from red blood cells by centrifugation at 1934 g (relative centrifugal force) for 20 min. Mononuclear cells were diluted 2:1 in pre-warmed RPMI cell culture medium supplemented with 20% autologous plasma and incubated over 24 h and 48 h. Viability (dye exclusion assay using Trypan Blue) of mononuclear cells was measured using a Neubauer counting chamber and ranged between 94.5 and 99.6%.

Due to the minimal-invasive collection and the fully anonymized processing of the samples, the local ethical commission (Ethics committee, Bayerische Landesärztekammer, Munich, Germany) decided that experiments can be performed in agreement with ethical standards and do not require an additional approval. All samples were obtained with informed consent. All experiments were performed in accordance with relevant guidelines and regulations.

### RNA extraction and quality control

RNA from mononuclear cells was isolated following the RNeasy Mini Kit (Qiagen). In brief, cells were lysed (RLT-buffer) and RNA precipitated by adding ethanol. Samples were transferred on a silica-membrane which binds RNA, washed three times, DNA was digested (DNase) on the column, samples washed again and RNA eluted in 60 µl RNase-free water.

The isolated *RNA* was quantified spectrophotometrically (NanoDrop, PeqLab Biotechnology, Erlangen, Germany). RNA integrity was assessed by 4200 TapeStation System (Agilent Technologies, Santa Clara, USA). Possible contamination by sample genomic DNA was controlled by PCR using primers for the actin gene. RNA specimens with a ratio of A260/A280 nm ≥ 2.0 and RNA integrity number (RIN) ≥ 8 were processed for qRT-PCR analysis.

### Real-Time quantitative reverse transcription polymerase chain reaction (qRT-PCR)

Aliquots of total RNA (e.g. 0.25 µg, 0.5 µg or 1 µg) were reverse transcribed with the High-Capacity cDNA Reverse Transcription Kit (Applied Biosystems, Life Technologies, Darmstadt, Germany). Equal amounts of template cDNA (10 ng per reaction for *FDXR* and *DDB2* and 50 ng per reaction for *WNT3* and *POU2AF1*) were used, mixed with the TaqMan Universal PCR Master Mix (Thermo Fisher Scientific Inc., Waltham, USA) and run using a QuantStudio 12 K OA Real-Time PCR System (Thermo Fisher Scientific Inc., Waltham, USA) as duplicate measurements. The numbers of inventoried and custom designed TaqMan assays (Thermo Fisher Scientific Inc., Waltham, USA) were 16 (*FDXR)*, 11 (*DDB2)*, 5 (*WNT3)* and 9 (*POU2AF1)*. The cycle threshold (Ct) values of the four genes were normalized relative to diluted (0.01 ng per reaction) 18S rRNA (Hs03003631_g1). Dilution of 18S rRNA represents an additional burden of that housekeeping gene, but unaltered 18S rRNA copy numbers after irradiation and similar copy numbers observed in different tissues and species outweigh this additional burden^19^. The ratio/fold-change (FC) relative to the unexposed sample at the same time point (reference) was determined by the delta-delta Ct-approach (FC = 2^(-∆∆Ct)^). A FC of one corresponds to a gene expression similar to unexposed samples. A FC higher or lower than one refers to a several-fold over- or under-expression of the gene of interest after exposure relative to the reference. All experimental work was performed according to the standard operating procedures implemented in our laboratory since 2008 when the Bundeswehr Institute of Radiobiology became DIN-certified by TÜV Süd München, Germany (DIN EN ISO 9001/2008).

### Data analysis

The following inclusion criteria were applied to select the most promising TaqMan assays for biodosimetry purposes:

1. Baseline (raw Ct-values) that were < 33 (up-regulated genes) and < 30 (down-regulated genes) were chosen to allow for a dose-dependent deregulation of gene expression within the linear dynamic range of qRT-PCR.
2. A difference in fold change of at least two additional units between three dose categories (0 Gy vs 0.5 Gy vs 4.0 Gy) was determined and a dose dependency above this value was assumed. For instance, if the radiation-induced differential gene expression after 0.5 Gy was tenfold over unexposed, the differential gene expression after 4 Gy irradiation had to be ≥ 12-fold. This cut-off represents a well-established value to adjust for methodological variance^10,20^.
3. GE changes should ideally persist over time (three days) to create a diagnostic time window^11,21^. Hence, a difference in fold change ± one unit between time points was determined. For instance, if the differential gene expression was 10.5-fold after 24 h (relative to unexposed), the differential gene expression after 48 h had to be between 9.5 and 11.5.
4. Inter-individual variability of the three donors was determined as a further characteristic of radiation-responsive exon-pairs. For this purpose, the sum of the standard deviations of the dose categories was calculated for each TaqMan assay.

### Statistical analysis

Descriptive statistics were performed using Excel. To examine the dose dependency of the inter-individual variability, repeated measurements analysis of variance (ANOVA) followed by Tukey’s test as a post-hoc test were used to compare the coefficient of variation (CV, standard deviation *100/mean) of the FCs. These analysis and graphical representations were performed using SPW (SigmaPlot, Version 14, Jandel Scientific, Erkrath, Germany).

## Results

### FDXR

**Figure 2:**
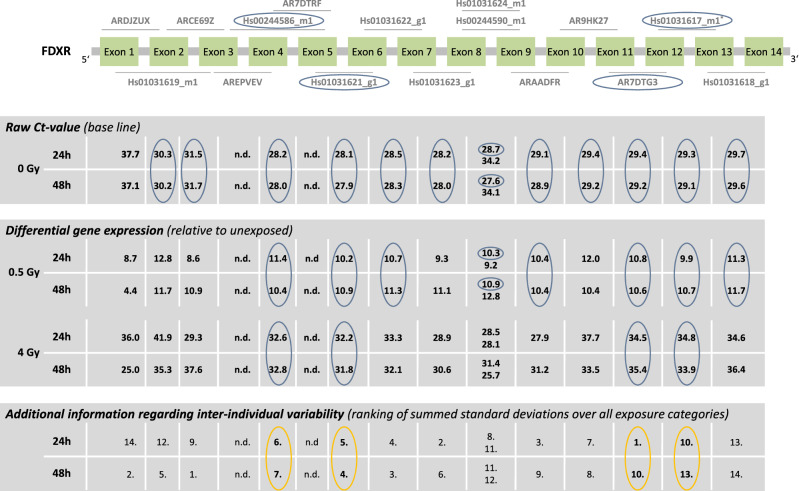
*FDXR* results. Display of TaqMan assay arrangement on a schematic image of *FDXR* (note: size ratio of exons to each other is not accurate), baselines and differential gene expression after 0.5 Gy and 4 Gy after 24 h and 48 h. At the bottom of the figure, the inter-individual variability is shown. The assay with the lowest sum of the standard deviation of the FCs over all dose categories is ranked 1st. Correspondingly the assay with the highest sum is ranked last. Starting with the baseline and working downwards, the assays meeting the criteria defined in the material and methods are circled. For illustration, the inter-individual variability of the TaqMan assays identified using the other criteria (baseline, dose dependency, time persistency) are circled in orange. The four encircled assays on the schematic image of *FDXR* are the ones most suitable for biodosimetry purposes. Asterisks mark the best coverage assay. Assay labels starting with the two capital letters “AR” can be found after creating an account at Thermo Fisher web site, entering the “Reorder Custom Assays” section with the implemented search function in the “Custom TaqMan Assay Design Tool”. *n.d.*  not detectable.

**Figure 3:**
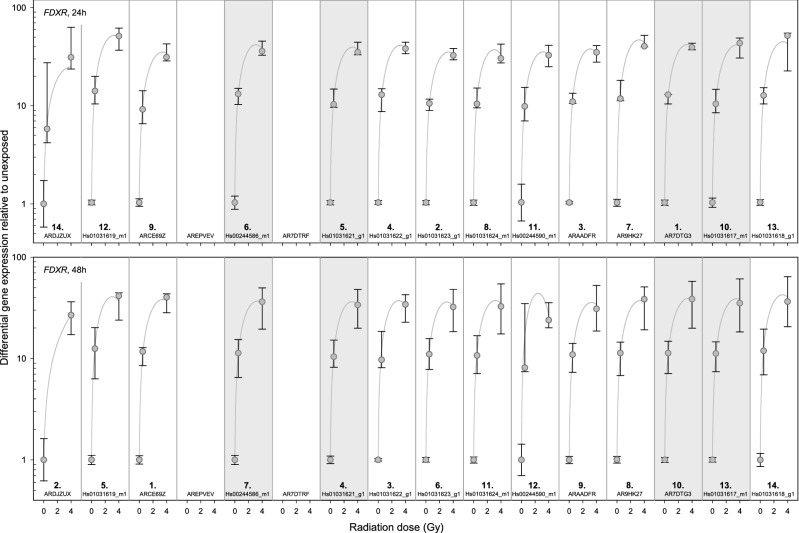
Inter-individual variability of *FDXR* TaqMan assays. Plotted is the differential gene expression relative to unexposed of *FDXR* for each assay after 24 h (upper panel) and 48 h (lower panel). The symbol represents the median, and the lower and upper whiskers represent the minimum and maximum fold change, respectively. Additionally plotted is the 2nd order regression line for each TaqMan assay. After summing up the standard deviations of radiation dose, ranks were assigned. 1st rank is the lowest inter-individual variability, 14th rank is the highest. The four assays highlighted in gray are those most suitable for biodosimetry as identified in Fig. 2 using other criteria (baseline, dose dependency, time persistency). Assay labels starting with the two capital letters “AR” can be found after creating an account at Thermo Fisher web site, entering the “Reorder Custom Assays” section with the implemented search function in the “Custom TaqMan Assay Design Tool”.

The baselines of all detectable TaqMan assays except for *Hs00244590_m1* and *ARJZUX* (raw Ct-values were 34–38) ranged between 28 and 32 (Fig. 2, supplementary table S1). Two TaqMan assays (*AREPVEV, AR7DTRF*) were not detectable (raw Ct-values > 40). Radiation-induced up-regulation of *FDXR* was detectable in all exon-regions without showing regional differences. Differential GE increased two- to threefold from 0.5 to 4 Gy, e.g., from tenfold (at 0.5 Gy) to 20–30-fold (at 4 Gy). For *Hs00244586_m1, Hs01031621_g1, AR7DTG3* and *Hs01031617_m1* a constant upregulation of GE was observed over time at 0.5 and 4 Gy. For the remaining exon-regions, a time dependent change in differential GE (difference in differential GE ≥ one unit) was observed either at 0.5 Gy and/or 4 Gy. *ARCE69Z* showed the least inter-individual variability overall (Fig. 3) and *Hs01031621_g1* showed the least inter-individual variability among the four TaqMan assays identified (Table 1) using the other criteria (baseline, dose dependency, time persistency). Baselines > 33 resulted in the largest inter-individual variabilities in sham-exposed samples (*Hs00244590_m1* and *ARDJZUX*).

**Table 1:** Results overview.

*Gene*	Hs.No	TaqMan assay characteristics								
		Baseline	FC			Time persistency	Dose dependency	Inter-individual variability (ranking in the)		
			0 Gy	0.5 Gy	4 Gy			Upper third	Middle third	Lower third
*FDXR*	Hs01031621_g1	28	1	11	32	✓	✓	✓		
	AR7DTG3	29	1	11	35	✓	✓	✓		
	Hs00244586_m1	28	1	11	33	✓	✓		✓	
	Hs01031617_m1	29	1	10	34	✓	✓			✓
*DDB2*	AR47X6H	27	1	5	10	✓	✓	✓		
	Hs03044951_m1	28	1	6	10	✓	✓		✓	
*WNT3*	Hs00902258_m1	30	1	0.6	0.2	✓	✓*	✓		
	Hs00902257_m1	30	1	0.6	0.2	✓	✓*	✓		
*POU2AF1*	Hs01573370_g1	23	1	0.8	0.4	✓	✓*	✓		
	Hs01573371_m1	24	1	0.8	0.4	✓	✓*	✓		

Displayed are the TaqMan assays per gene that were most suitable for biodosimetry purposes considering adequate baseline, the magnitude of radiation-induced differential gene expression, similar gene expression over time and inter-individual variability. Asterisks represent a dose-dependency only for 4 Gy. Assay labels starting with the two capital letters “AR” can be found after creating an account at Thermo Fisher web site, entering the “Reorder Custom Assays” section with the implemented search function in the “Custom TaqMan Assay Design Tool”. FC = fold change with the reference 0 Gy set to a FC of 1.

### DDB2

**Figure 4:**
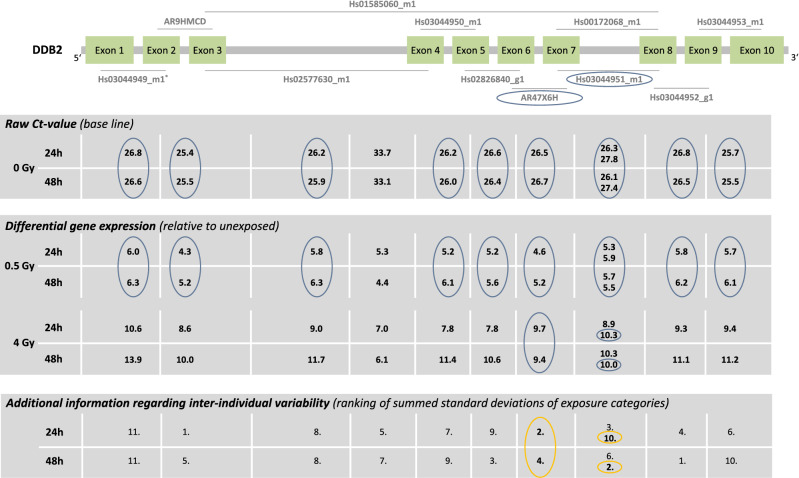
*DDB2* results. Display of TaqMan assay arrangement on a schematic image of *DDB2* (note: size ratio of exons to each other is not accurate), baselines and differential gene expression after 0.5 Gy and 4 Gy after 24 h and 48 h. At the bottom of the figure, the inter-individual variability is shown. The assay with the lowest sum of the standard deviation of the FCs over all dose categories is ranked 1st. Correspondingly the assay with the highest sum is ranked last. Starting with the baseline and working downwards, the assays meeting the criteria defined in the material and methods are circled. For illustration, the inter-individual variability of the TaqMan assays identified using the other criteria (baseline, dose dependency, time persistency) are circled in orange. The two encircled assays on the schematic image of *DDB2* are the ones most suitable for biodosimetry purposes. Asterisks mark the best coverage assay. Assay labels starting with the two capital letters “AR” can be found after creating an account at Thermo Fisher web site, entering the “Reorder Custom Assays” section with the implemented search function in the “Custom TaqMan Assay Design Tool”.

**Figure 5:**
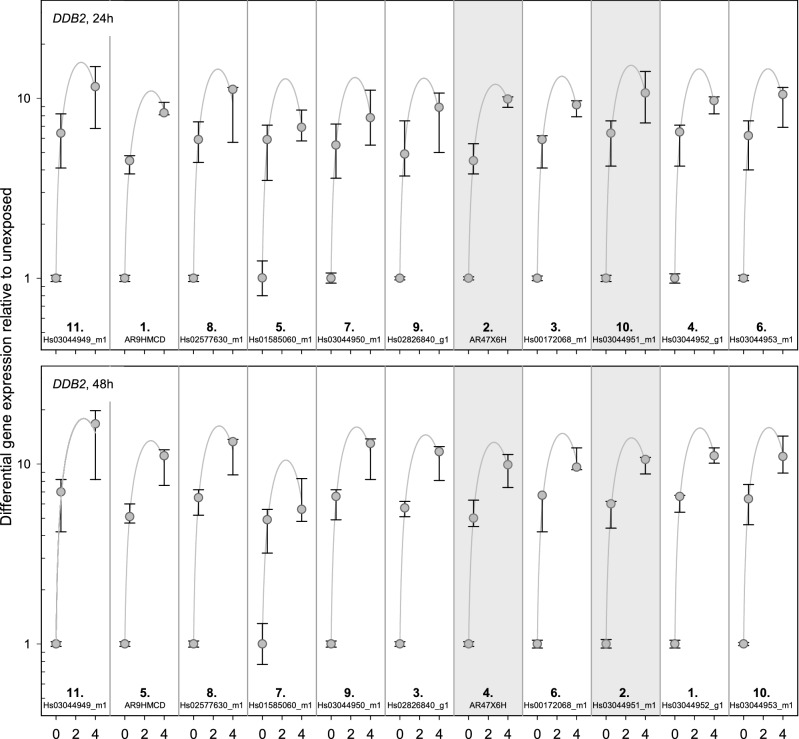
Inter-individual variability of *DDB2* TaqMan assays. Plotted is the differential gene expression relative to unexposed of *DDB2* for each assay after 24 h (upper panel) and 48 h (lower panel). The symbol represents the median, and the lower and upper whiskers represent the minimum and maximum fold change, respectively. Additionally plotted is the 2nd order regression line for each TaqMan assay. After summing up the standard deviations of radiation dose, ranks were assigned. 1st rank is the lowest inter-individual variability, 11th rank is the highest. The two assays highlighted in gray are those most suitable for biodosimetry as identified in Fig. 4 using other criteria (baseline, dose dependency, time persistency). Assay labels starting with the two capital letters “AR” can be found after creating an account at Thermo Fisher web site, entering the “Reorder Custom Assays” section with the implemented search function in the “Custom TaqMan Assay Design Tool”.

The baselines of all used TaqMan assays except for *Hs01585060_m1* (raw Ct-values were 33–34) ranged between 25 and 28 (Fig. 4). All exon-regions showed sufficient up-regulation of GE without any regional differences. For *AR47X6H* and *Hs03044951_m1* a constant up-regulation of GE was observed over time at 0.5 Gy and 4 Gy. For the remaining exon-regions, a time dependent change in differential GE (difference in differential GE ≥ one unit) was observed either at 0.5 Gy and/or 4 Gy. *AR9HMCD* showed the least inter-individual variability overall (Fig. 5) and *AR47X6H* showed the least inter-individual variability among the two TaqMan assays (Table 1) identified using the other criteria (baseline, dose dependency, time persistency). A baseline > 33 resulted in the largest inter-individual variability in sham-exposed samples in *Hs01585060_m1*.

### WNT3

**Figure 6:**
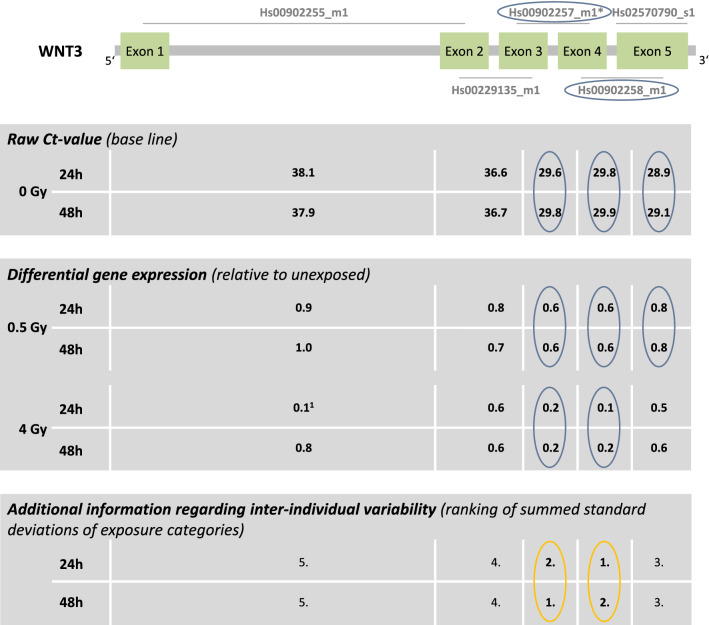
*WNT3* results. Display of TaqMan assay arrangement on a schematic image of *WNT3* (note: size ratio of exons to each other is not accurate), baselines and differential gene expression after 0.5 Gy and 4 Gy after 24 h and 48 h. At the bottom of the figure, the inter-individual variability is shown. The assay with the lowest sum of the standard deviation of the FCs over all dose categories is ranked 1st. Correspondingly the assay with the highest sum is ranked last. Starting with the baseline and working downwards, the assays meeting the criteria defined in the material and methods are circled. For illustration, the inter-individual variability of the TaqMan assays identified using the other criteria (baseline, dose dependency, time persistency) are circled in orange. The two encircled assays on the schematic image of *WNT3* are the ones most suitable for biodosimetry purposes. Asterisks mark the best coverage assay. Data from only one donor, for the other two donors the assay was not detectable^1^.

**Figure 7:**
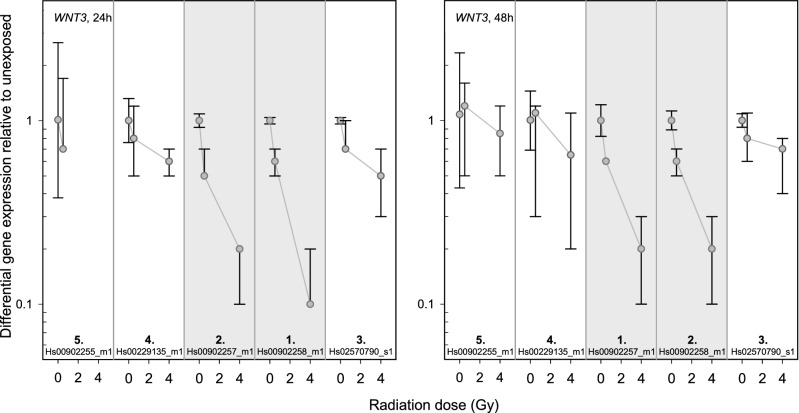
Inter-individual variability of *WNT3* TaqMan assays. Plotted is the differential gene expression relative to unexposed of *WNT3* for each assay after 24 h (left panel) and 48 h (right panel). The symbol represents the median, and the lower and upper whiskers represent the minimum and maximum fold change, respectively. Additionally, symbols are connected with simple straight lines for each TaqMan assay. After summing up the standard deviations of radiation dose, ranks were assigned. 1st rank is the lowest inter-individual variability, 5th rank is the highest. The two assays highlighted in gray are those most suitable for biodosimetry as identified in Fig. 6 using other criteria (baseline, dose dependency, time persistency).

The baselines of the three TaqMan assays located at the 3’-end of the gene ranged between 29 and 30 Ct-values (Fig. 6), whereas the baselines of the remaining two TaqMan assays located at the 5’-end of *WNT3* ranged between 37 and 38 Ct-values. Hence, only the exon-regions at the 3’-end of the gene covered by *Hs00902257_m1* and *Hs00902258_m1* showed a sufficient down-regulation of GE at 4 Gy. For *Hs00902257_m1* and *Hs00902258_m1* a constant down-regulation of GE was seen over time at 0.5 Gy and 4 Gy. Both (*Hs00902257_m1* and *Hs00902258_m1*) showed the lowest inter-individual variability over all examined exon-pairs (Fig. 7, Table 1). Baselines > 33 resulted in the largest inter-individual variabilities in sham-exposed samples (*Hs00902255_m1* and *Hs00229135_m1*).

### POU2AF1

**Figure 8:**
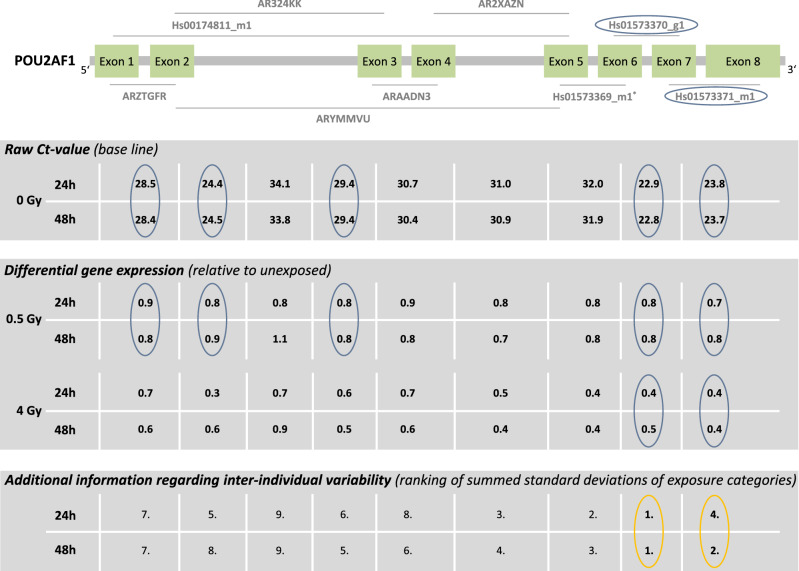
*POU2AF1* results. Display of TaqMan assay arrangement on a schematic image of *POU2AF1* (note: size ratio of exons to each other is not accurate), baselines and differential gene expression after 0.5 Gy and 4 Gy after 24 h and 48 h. At the bottom of the figure, the inter-individual variability is shown. The assay with the lowest sum of the standard deviation of the FCs over all dose categories is ranked 1st. Correspondingly the assay with the highest sum is ranked last. Starting with the baseline and working downwards, the assays meeting the criteria defined in the material and methods are circled. For illustration, the inter-individual variability of the TaqMan assays identified using the other criteria (baseline, dose dependency, time persistency) are circled in orange. The two encircled assays on the schematic image of *POU2AF1* are the ones most suitable for biodosimetry purposes. Asterisks mark the best coverage assay. Assay labels starting with the two capital letters “AR” can be found after creating an account at Thermo Fisher web site, entering the “Reorder Custom Assays” section with the implemented search function in the “Custom TaqMan Assay Design Tool”.

**Figure 9:**
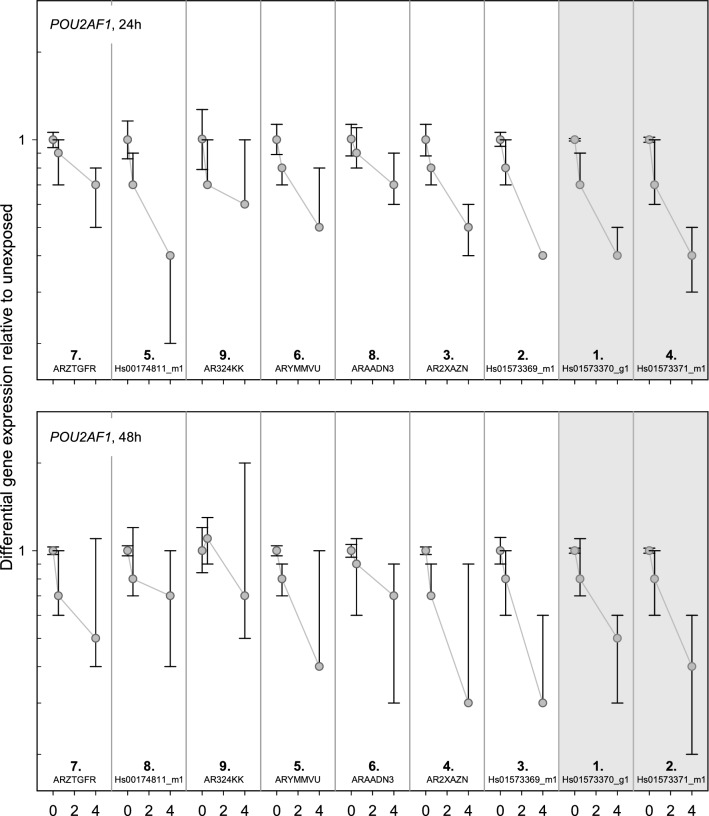
Inter-individual variability of *POU2AF1* TaqMan assays. Plotted is the differential gene expression relative to unexposed of *POU2AF1* for each assay after 24 h (upper panel) and 48 h (lower panel). The symbol represents the median, and the lower and upper whiskers represent the minimum and maximum fold change, respectively. Additionally symbols are connected with simple straight lines for each TaqMan assay. After summing up the standard deviations of radiation dose, ranks were assigned. 1st rank is the lowest inter-individual variability, 9th rank is the highest. The two assays highlighted in gray are those most suitable for biodosimetry as identified in Fig. 6 using other criteria (baseline, dose dependency, time persistency). Assay labels starting with the two capital letters “AR” can be found after creating an account at Thermo Fisher web site, entering the “Reorder Custom Assays” section with the implemented search function in the “Custom TaqMan Assay Design Tool”.

The baselines of five TaqMan assays (*ARZTGFR, Hs00174811_m1, ARYMMVU, Hs01573370_g1, Hs01573371_m1*) ranged between 23 and 29 Ct-values (Fig. 8). For the remaining TaqMan assays the baseline Ct-values were above 30. Only *Hs01573370_g1* and *Hs01573371_m1* at the 3’-end of the gene showed sufficient down-regulation of GE at 4 Gy and a constant down-regulation of GE over time. Both assays (*Hs01573370_g1* and *Hs01573371_m1*) showed the lowest inter-individual variability overall (Fig. 9, Table 1). A baseline > 33 resulted in the largest inter-individual variability in sham-exposed samples in *AR324KK*.

### Inter-individual variability—dose dependency

**Figure 10:**
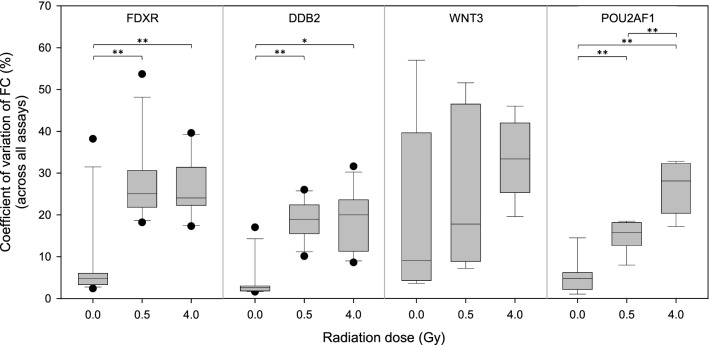
Box plots of coefficient of variations of fold changes of *FDXR, DDB2, WNT3, POU2AF1* across all assays per dose category. Horizontal lines within boxplots represent medians. The upper and lower whiskers correspond to 1.5 × of the interquartile range. Black dots represent outliers. *p*-Values were calculated between exposure groups (0 Gy, 0.5 Gy, 4 Gy) using repeated measurements ANOVA with Tukey’s Test as a post-hoc test and *p*-values ≤ 0.01 and ≤ 0.001 are marked with one and two asterisks. *ANOVA* analysis of variance.

In all four genes the inter-individual variability increased after radiation exposure (Fig. 10). For *FDXR, DDB2* and *POU2AF1* there was a statistically significant (*p* ≤ 0.002) difference of the CV of the FCs across all exon-pairs regarding the 0.5 Gy and 4 Gy irradiated samples relative to the sham irradiated control. For *POU2AF1* there was additionally a statistically significant difference (*p* < 0.001) of the CVs of the FCs across all exon-pairs with respect to the 0.5 Gy and 4 Gy irradiated samples. Only for *WNT3* there were no statistical differences observed. However, when comparing the medians of the dose categories of *WNT3*, a tendency toward greater inter-individual variability was observed at higher radiation doses. For all genes studied, inter-individual variability increased three to sixfold due to irradiation.

## Discussion

GE analysis was recently reported to be suitable for triage (unexposed vs low- and high exposed) and for biodosimetry purposes after radiation exposure, particularly when considering a set of four genes (*FDXR, DDB2, WNT3* and *POU2AF1*)^8^. Even so, methodologic differences rendered some results that differed across teams during international exercises^16^. These might be explained by employment of different TaqMan assays targeting different exon-pairs. Therefore, the aim of this study was to improve detection of GE fold changes by identifying the most radiation-responsive exon-regions of these genes.

Our results indicate marginal differences between TaqMan assays for *FDXR* and *DDB2*, genes often used in biodosimetry and effect prediction, because radiation-induced regulations are comparable over all exon-regions of both genes, which was opposite with what we found for *POU2AF1* and *WNT3* that showed radiation-induced downregulation only at exon-pairs on the 3’ end. Other factors, such as varying RNA quantity/quality, other housekeeping genes or differences in qRT-PCR data analysis using other baselines and thresholds, might have had an impact on differences among laboratories of these international exercises, although in theory they should not. Also, differences in dose estimates deduced from calibration curves might be caused when applying different regression models. In the context of the RENEB 2021 inter-laboratory comparison exercise, the impact of these other factors is currently under investigation (unpublished results).

Another aim of this study, was to evaluate the most promising exon-assays that could be used for biodosimetry and health effect prediction. For this purpose, we considered an appropriate baseline for gene detection, the magnitude of radiation-induced differential GE, inter-individual variability and gene expression persistency over time. Following these criteria, we identified several promising TaqMan assays for biodosimetry purposes. Assays ending in “_m1” are generally preferred, as “_g1” assays can also detect genomic DNA^18^. We found that the so called “best coverage assays” as recommended by the manufacturer for qRT-PCR analysis did not necessarily perform best in the present radiation exposure study. For *FDXR* and *WNT3* the best coverage assays were among the ones suitable for biodosimetry purposes. However, *Hs00172068_m1 (DDB2)* showed no GE persistency over 48 h after 4 Gy irradiation, and a mean baseline of 32 Ct-values was too low for *Hs01573369_m1 (POU2AF1)* to allow radiation-induced down-regulation of GE (resulting in increased Ct-values) in the linear dynamic range of qRT-PCR. Here, other assays were superior to the best coverage TaqMan assays. Clearly, the best coverage assay does not automatically fulfill all the criteria needed for a good GE biomarker used for biodosimetry or health effect prediction purposes.

The existence of radiation-responsive exons has been reported over the last decade using different types of biologic materials (PBMC, fibroblasts, lymphoblastoid cell line)^22–24^. Radiation-responsive exons have been identified depending on the genes studied *(CDKN1A, DDB2)* and support our *POU2AF1* and *WNT3* findings. Alternatively, some genes showed no difference in the level of radiation-induced gene expression between exon-regions *(EDA2R)*^23,24^*,* which is in line with our *FDXR* and *DDB2* results. However, Macaeva et al. described significant differences in the gene expression of *FDXR* for different exons in qRT-PCR 8 h after radiation exposure. This contrasts to our results, but different time points after irradiation and examinations of all *FDXR* exons after high radiation exposures in our study might explain the discrepancies. Notably, the prediction of later developing health effects based on early gene expression changes as presented in our study is complementary, but differently, to a biodosimetric approach. If radiation-responsive exon-regions exist, then one would expect to see a dissimilar differential GE at the exon level compared to the gene level. Recently reported difficulties in validating NGS data of some but not all genes (summation of reads across whole genes) vs. using qRT-PCR (selective targeting of exon-regions) provide further evidence for the existence of radiation-responsive exon-regions^17^. Reanalyzing NGS-data at the exon level and rerunning qRT-PCR with TaqMan assays covering precisely these exon regions led to an agreement of NGS-data with qRT-PCR^17^. Validation of NGS data with qRT-PCR, the gold-standard GE methodology, bears challenges: In NGS, read lengths can be as short as 36 base pairs (bp) and, in addition, sequencing errors occur. A unique genomic mapping is thus not always possible^25^. The assay sequence length in qRT-PCR contains at least 60 bp and is therefore unambiguous for a specific location in the genome. Furthermore, NGS and qRT-PCR, are affected by single nucleotide polymorphisms (SNPs). In NGS, SNPs can lead to mapping errors, distorting NGS results. In qRT-PCR SNPs can prohibit the development of an amplification plot and therefore cause undetectable Ct-values, making validation of NGS data difficult.

For an exon approach in qRT-PCR, the use of the nucleotide sequence of two adjacent exons (exon spanning probe design) as the TaqMan assay sequence represents a common standard. Placing the forward and reverse primer and probe exclusively in one exon is possible but not recommended due to the likely detection of genomic DNA^26^. Alternative 5’ and 3’ splicing sites (supplementary Fig. S1) within exons are also a limitation of this approach, exemplified in the present study by *AR7DTRF* and *AR7DTRF (FDXR,* supplementary Fig. S2). Moreover, our exon approach does not allow us to conclude about the transcript level of these four genes, e.g. which transcript is detected, the transcript abundance or the induction of specific transcripts after irradiation. Hence, this additional information regarding the transcript level is missing. However, it does not render our study on identification of most suitable radiation-responsive exon-pairs for biodosimetry and in particular for prediction of acute health effects uninformative. In the instance of *WNT3* a gene with only one known transcript was studied. The described differences in differential GE could therefore be due to transcript variants not yet described or due to differences in PCR reaction efficiency of different assays and not due to differences in expression of different exon-pairs. However, other than SYBR-green chemistry, more expensive TaqMan assays comprise already validated primer–probe designs, so that the quantitative ΔΔ-Ct-approach can be applied. Also, raw Ct-values of baseline (0 Gy) gene expression measurements are in most instances and in particular for the recommended assays (Table 1) comparable over almost all examined exons, which argues against differences in PCR reaction efficiency inherent to the employed TaqMan assays.

Transcript variants are of interest when novel splicing sites are specifically induced by irradiation and can be used as biomarkers for radiation exposure^23,27^. To date, only nanopore technology allows detection of specific transcript variants. For example *FDXR-218* and *FDXR-219* (Ensemble database) using nanopore technology detected no counts in control samples compared to irradiated samples, which showed a significant increase in counts^27^.

In another study, we examined baseline inter-individual variabilty on 200 unirradiated human samples^28^. Median standard deviation values—as a reflection of inter-individual variability—of our four genes in this study were comparable to these published data. However, in the current study we demonstrated a three to sixfold increase in inter-individual variability after radiation exposure. Nevertheless, using the TaqMan assays we identified by considering our four criteria (baseline, dose dependency, time persistency, inter-individual variability, Table 1), we observed a nearly complete separation between the three exposure groups (unexposed, low-exposed, high-exposed). Specifically, combining the results of the examined genes resulted in complete discrimination of the exposure groups, thus, confirming previous findings^11,12^ related to the diagnostic significance of this gene set.

Study limitations include a small sample size. However, the results are similar among all examined individuals, which argues against findings by chance. Nevertheless, these results need to be validated in larger studies. The usage of lithium-heparin tubes for blood collection instead of EDTA is also a minor limitation of this study. In a previous inter-laboratory comparison study, we demonstrated that using lithium-heparin coated vials resulted in slightly higher raw Ct-values, e.g., from Ct-value of 20–21^29^. Also, raw Ct-values of our genes were well within the linear-dynamic range of the method, so that a slight shift to higher Ct-values would not alter our results. Hence, both, EDTA and lithium-heparin are suitable for examining the radiation induced gene expression changes examined within this study. Furthermore, this study represents the most comprehensive study on often used genes for biodosimetry and ARS prediction on an exon-level of these four genes.

In summary, a comparable induction of GE after irradiation observed in most *FDXR* and *DDB2* exon-regions does not explain huge differences in dose estimates reported by different teams in large biodosimetry exercises. Exon-related GE differences in *POU2AF1* and *WNT3* underline difficulties in validation of NGS data using qRT-PCR. Using several criteria such as baseline, magnitude of radiation-induced differential GE, time persistency and inter-individual variability, we identified several TaqMan assays that could be used for biodosimetry purposes and identification of clinically relevant groups.

## Supplementary Information

Supplementary Figure 1.

Supplementary Figure 2.

Supplementary Table 1.
