# Supplementary material for: Identifying radiation responsive exon-regions of genes often used for biodosimetry and acute radiation syndrome prediction
Source: Sci Rep. 2022 Jun 9;12:9545. doi: 10.1038/s41598-022-13577-4 (PMC9184472; doi:10.1038/s41598-022-13577-4)
Supplement: Supplementary file 1 — Supplementary Figure 1. [file 41598_2022_13577_MOESM1_ESM.pdf]

Supplementary figure 1
Schüle et al.

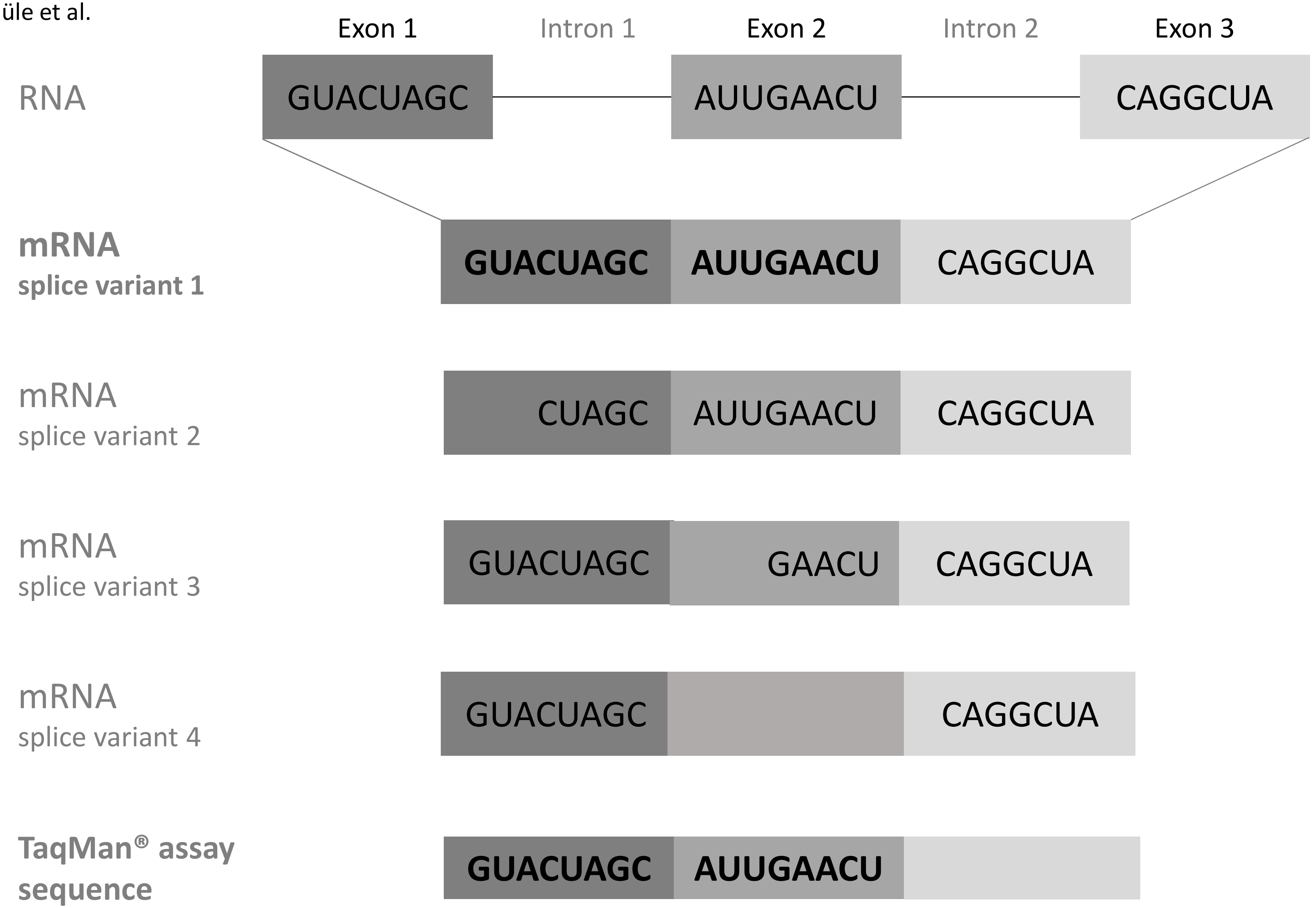

Supplementary figure 1: Influence of splicing variants on assay design. Schematic illustration of four different mRNA splicing variants on grounds of alternative splicing sites based on a fictional RNA sequence. The assay sequence in bold on the bottom only covers, due to its design, splicing variant 1 (also written in bold). The assay does not map splicing variants 2-4.
