# Supplementary material for: Identifying radiation responsive exon-regions of genes often used for biodosimetry and acute radiation syndrome prediction
Source: Sci Rep. 2022 Jun 9;12:9545. doi: 10.1038/s41598-022-13577-4 (PMC9184472; doi:10.1038/s41598-022-13577-4)
Supplement: Supplementary file 2 — Supplementary Figure 2. [file 41598_2022_13577_MOESM2_ESM.pdf]

Supplementary figure 2

Schüle et al.

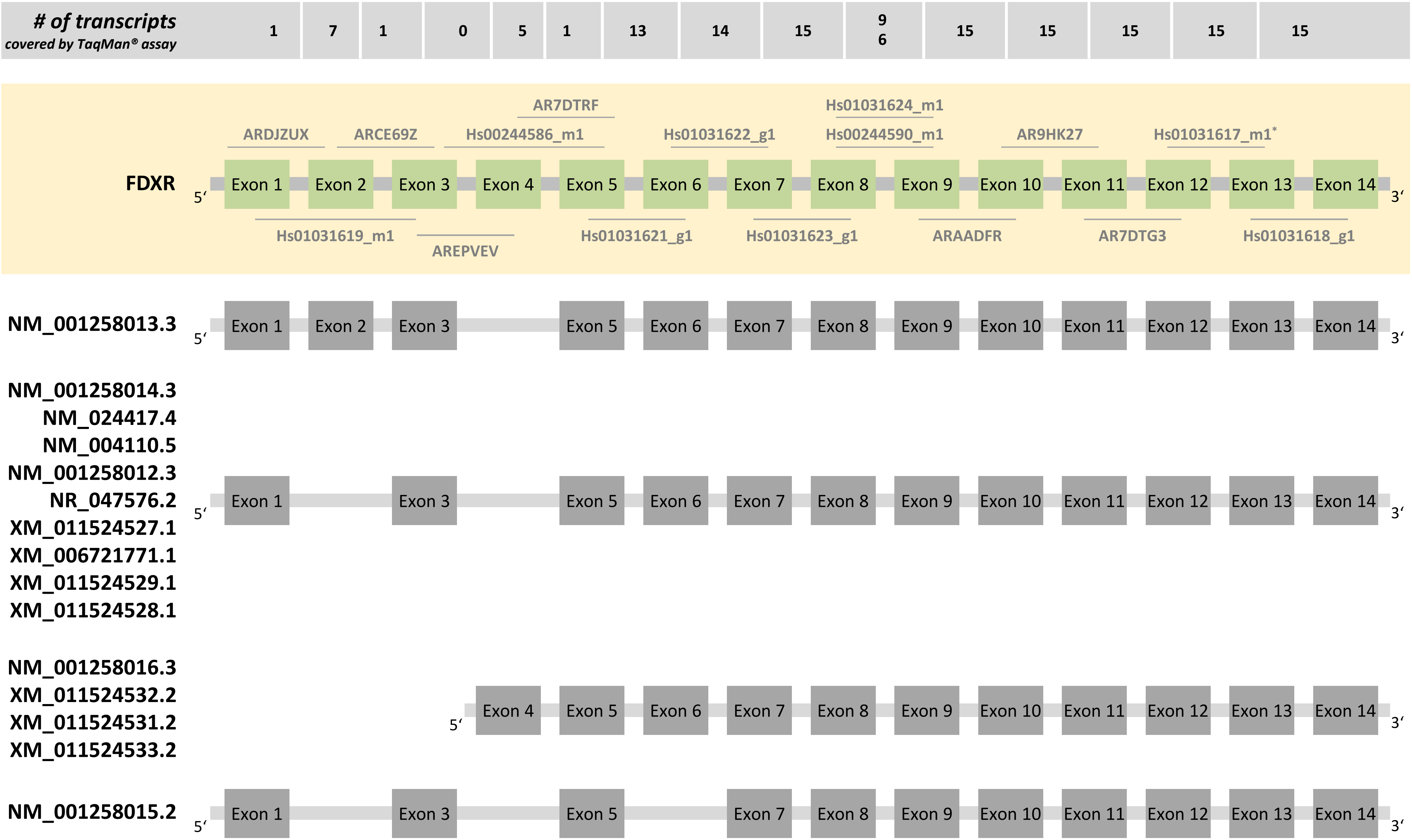

Supplementary figure 2: Schematic illustration of *FDXR*, its transcripts and the number of transcripts covered by the examined TaqMan® assay according to RefSeq database. *AR7DTRF* developed no amplification plot, which might be caused by an exon primer-probe design recognizing only one low-abundance transcript. *AREPVEV* is not detectable, because no known transcript exists, which contains exon 3 and 4. # = number of transcripts. Assay labels starting with the two capital letters "AR" can be found after creating an account at Thermo Fisher web site, entering the "Reorder Custom Assays" section with the implemented search function in the "Custom TaqMan® Assay Design Tool.
